# The Role of Group 3 Innate Lymphoid Cells in Lung Infection and Immunity

**DOI:** 10.3389/fcimb.2021.586471

**Published:** 2021-02-25

**Authors:** Dan Yang, Xinning Guo, Tingxuan Huang, Chuntao Liu

**Affiliations:** Department of Respiratory and Critical Care Medicine, West China School of Medicine and West China Hospital, Sichuan University, Chengdu, China

**Keywords:** innate immunity, Group 3 innate lymphoid cells, lung infections, airway inflammation, cytokines, interleukin-22, interleukin-17

## Abstract

The lung is constantly exposed to environmental particulates such as aeroallergens, pollutants, or microorganisms and is protected by a poised immune response. Innate lymphoid cells (ILCs) are a population of immune cells found in a variety of tissue sites, particularly barrier surfaces such as the lung and the intestine. ILCs play a crucial role in the innate immune system, and they are involved in the maintenance of mucosal homeostasis, inflammation regulation, tissue remodeling, and pathogen clearance. In recent years, group 3 innate lymphoid cells (ILC3s) have emerged as key mediators of mucosal protection and repair during infection, mainly through IL-17 and IL-22 production. Although research on ILC3s has become focused on the intestinal immunity, the biology and function of pulmonary ILC3s in the pathogenesis of respiratory infections and in the development of chronic pulmonary inflammatory diseases remain elusive. In this review, we will mainly discuss how pulmonary ILC3s act on protection against pathogen challenge and pulmonary inflammation, as well as the underlying mechanisms.

## Introduction

The lung is constantly exposed to particulates such as aeroallergens, pollutants, or microorganisms from the external environment. Maintenance of respiratory epithelial barrier function is crucial to limit exposure to environmental stimuli and pathogens (Sonnenberg et al., 2010a). Two emerging T helper (Th) cell subsets, Th17 and Th22 cells, and their respective prototype cytokines interleukin 17A (IL-17A) and IL-17F and IL-22, link the immune response to tissue inflammation, and play important roles in host defense, autoimmunity, and allergic diseases (Akdis et al., 2012). IL-22 and IL-17 are coexpressed not only by these cells, but also by the subsets of innate-like lymphocytes, such as group 3 innate lymphoid cells (ILC3s) (Takatori et al., 2009; Raifer et al., 2012). Here we will highlight recent advances on how IL-17^+^/IL-22^+^ ILC3s participate in homeostasis and immune-regulation in the lung, as well as their role in lung infectious diseases and airway immunity.

## Group 3 Innate Lymphoid Cells

Innate lymphoid cells (ILCs) are a population of immune cells found in a variety of tissue sites, particularly barrier surfaces such as the lung, the intestine, the skin, and mucosal membranes. ILCs are divided into five subsets depending on expression of the indicated transcription factors involved in their development and different cytokine-producing capacity (Cerwenka and Lanier, 2016; Melo-Gonzalez and Hepworth, 2017; Vivier et al., 2018; Borger et al., 2019). The development and function of ILCs are shown in **Supplementary Figure 1**.

While all ILC subsets have been identified in the respiratory system, the majority of research studies to date have focused on ILC2s, especially in the allergic airway inflammation (AAI) (Scanlon and McKenzie, 2012; Halim and McKenzie, 2013; Bernink et al., 2014; Aron and Akbari, 2017; Mindt et al., 2018; Kato, 2019). In recent years, ILC3s in the innate immune system have attracted more and more attention for their involvement in the maintenance of mucosal homeostasis, inflammation regulation, tissue remodeling and pathogen clearance. ILC3s are one of the ILC subgroups, characterized by the expression of retinoic acid receptor-related orphan receptor *γt* (ROR*γ*t) for lineage specification. ILC3s produce cytokines including IL-17A, IL-17F, IL-22, and granulocyte-macrophage colony-stimulating factor (GM-CSF) upon stimulation with myeloid or granulocyte cell-derived cytokines such as IL-1β and IL-23 (Hubeau et al., 2001; Cupedo et al., 2009; Buonocore et al., 2010; Hughes et al., 2010; Pearson et al., 2016). Adult ILC3s can be further subdivided by the expression of natural killer (NK) cells-associated receptor specifically NKp46, namely natural cytotoxicity receptor (NCR), in both human and mice (Satoh-Takayama et al., 2008; Luci et al., 2009; Sanos et al., 2009), with the addition of NKp44 in human (Hoorweg et al., 2012; Glatzer et al., 2013).

Discrepancies exist in the characterization of the (human and mouse) ILC subsets in different tissues (Annunziato et al., 2015; Montaldo et al., 2015; Vivier et al., 2018; Yudanin et al., 2019). The frequency and number of ILC3s reported in human and murine lungs vary greatly between studies. As for human, De Grove et al. reported ILC3s accounted for almost two thirds of ILCs in the lungs of patients with solitary pulmonary tumors (De Grove et al., 2016). NK cells and ILC3s were detected as the most prevalent group in human lungs (De Grove et al., 2016; Simoni et al., 2017), while Yudanin et al. reported that ILC2s were dominant in murine lungs (Yudanin et al., 2019). In the study by Van Maele et al., ROR*γ*t^+^ ILC3s account for 30% of the total ILCs in the murine lungs, most of which coexpress C-C motif chemokine receptor 6 (CCR6, the receptor for the epithelial chemokine CCL20) (Van Maele et al., 2014). There are approximately 2,000 to 22,000 pulmonary ILC3s in the wide-type mice in various studies (Kim et al., 2014; Van Maele et al., 2014; Everaere et al., 2016; Yanagisawa et al., 2017); however, the number reported by Ardain et al. is far smaller than the other studies in the *Mycobacterium tuberculosis* (Mtb)-infected murine lungs (Ardain et al., 2019) and in the murine bronchoalveolar lavage fluid (BALF) (Warren et al., 2019). There might be many reasons for the discrepancies, for example, biology and characterization differences of ILC3s within the lungs and airways between species, and not exactly the same cell populations defined in various studies with diverse markers.

## Lung ILC3s Activation and Function

The key role of IL-23 and IL-1β in ILC3 activation has been confirmed in lung infection (Van Maele et al., 2014; Muir et al., 2016; Trevejo-Nunez et al., 2016; Ardain et al., 2019) and airway inflammatory diseases (Kim et al., 2014; Everaere et al., 2016; Shikhagaie et al., 2017; Yanagisawa et al., 2017; Komlosi et al., 2018; Kim et al., 2019; Lee et al., 2020). Dendritic cells (DCs) in the intestinal mucosa secrete IL-23 and/or IL-1β to stimulate cytokine production of ILC3s in response to pathogens or commensals (Van Maele et al., 2010; Kinnebrew et al., 2012; Satpathy et al., 2013; Gray et al., 2017). Gray et al. have demonstrated that intestinal DCs capture antigen from commensal bacteria and induce CCR4 expression on ILC3s, which direct their postnatal trafficking to the murine lungs (Gray et al., 2017). Their results established that postnatal colonization by intestinal commensal bacteria could mediate lung mucosal immunity, which was pivotal in the development of lung defenses in mice. In 2014, Kim et al. for the first time demonstrated the signaling pathway of ILC3 activation in a mouse model with a high-fat diet (HFD)-induced airway hyperreactivity (AHR). The HFD promotes the proliferation of IL-17A^+^ ILC3s by inducing macrophages to secrete IL-1β *via* activation of the NLRP3 inflammasome, suggesting involvement of an NLRP3-IL-1β-ILC3-IL-17A axis (Kim et al., 2014). In a murine infectious model, IL-22-producing CCR6^+^ ILC3s were rapidly accumulated in the lung upon IL-23 stimulation, in a DC-triggered and MyD88-dependent manner (Cai et al., 2019).

In addition to these two most common cytokines reported, IL-2 can also amplify the IL-17A production of murine lung ILC3s (Muir et al., 2016; Kim et al., 2019), but specific mechanism has not been investigated in these studies. Recently, Xiong et al. reported that inflammatory monocytes (IMs) were activated and promptly recruited to the lungs and produce tumor necrosis factor (TNF), facilitating the rapid accumulation of CCR6^+^ ILC3s (Xiong et al., 2016). Furthermore, engagement of aryl hydrocarbon receptor (AhR), a metabolite-sensing nuclear receptor, might also promote ILC3 postnatal expansion and IL-22 production (Kiss et al., 2011; Lee et al., 2011; Qiu et al., 2012; Gronke et al., 2019). These years, more and more studies are exploring the specific sources of the cytokines and the possible signals through which they induce the expansion and/or recruitment of lung ILC3s in murine. However, the potential mechanisms in humans remain poorly understood, and research studies are warranted in the future.

**Figure 1:**
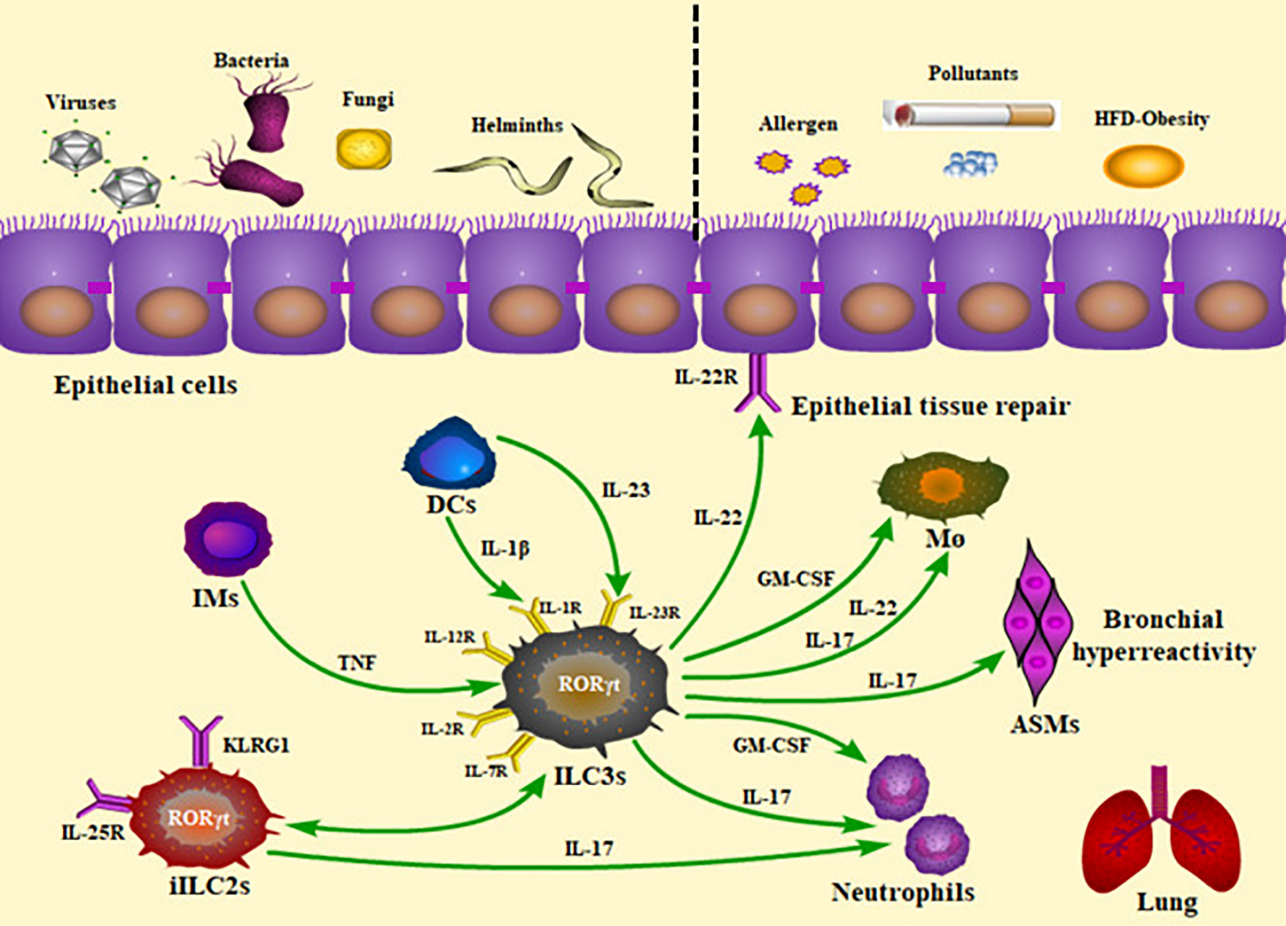
Roles of ILC3s and the production of cytokines in the lung inflammatory and infectious diseases. The presence of ILC3s within the lung and their rapid production of cytokines allow them to play a critical role in homeostasis, infection, and inflammation. Pulmonary ILC3s act on protection against viral, bacterial, parasitic, and fungal challenge, as well as contribution to airway inflammation. The panel showed the mechanisms underlying the ILC3-mediated immunity. IMs, inflammatory monocytes; DCs, dendritic cells; iILC2s, inflammatory ILC2s; TNF, tumor necrosis factor; HFD, high-fat diet; ILC3s, group 3 innate lymphoid cells; ASMs, airway smooth muscles; M ø, macrophages; GM-CSF, granulocyte-macrophage colony stimulating factor.

Although both cytokines are essential in the host defense at mucosal surfaces during bacterial infection, IL-17 mainly augments inflammation, whereas IL-22 usually plays a tissue-protective role, by maintaining epithelial barrier integrity and tissue homeostasis, preventing lung fibrosis and reducing airway inflammation (Dudakov et al., 2015; Valeri and Raffatellu, 2016; Felton et al., 2018; Fukaya et al., 2018; Lo et al., 2019). IL-17A may regulate the expression of IL-22 and promote proinflammatory functions of IL-22 in airway damage and inflammation (Andoh et al., 2005; Boniface et al., 2005; Sonnenberg et al., 2010b). In murine lungs, IL-22 is mainly produced by ILC3s in addition to Th17 cells and *γδ* T cells (Van Maele et al., 2014); in human lungs, ILC3s are primary sources of IL-22 in the newborns (Gray et al., 2017), while NK cells (Xu et al., 2014), Th17 cells (Sonnenberg et al., 2010b), and *γδ* T cells (Simonian et al., 2010) have been reported as the principal sources in adults. Rapid secretion of IL-17A and IL-22 highlights the role of ILC3s in lung diseases, as the IL-17/IL-22 axis contributes a lot to the epithelial immunity (**Figure 1**). Interestingly, De Grove also observed GM-CSF production of human pulmonary ILC3s (De Grove et al., 2016). Since GM-CSF-producing ILC3s were demonstrated in the mouse intestine which contributed to T-cell homeostasis (Longman et al., 2014; Mortha et al., 2014), exploring the findings further would be valuable.

Moreover, in recent years, it has been found that plasticity existed in ILC subsets. These populations share common progenitors and transcription factors and can communicate with the surrounding microenvironment and may be preprogrammed with the cytokine production profiles based on their plasticity (Montaldo et al., 2015; Bal et al., 2020). ST2 and IL-17RB are receptors for IL-33 and IL-25, respectively. IL-33 and IL-25 elicit two populations of ILC2s; the former boosts Lin^−^IL-17RB^low^KLRG1^int^ST2^+^ natural ILC2s (nILC2s) in the lung, whereas the latter accumulates Lin^−^IL-17RB^high^KLRG1^high^ST2^−^ inflammatory ILC2 (iILC2s) (Huang et al., 2015). IL-25-responsive ilLC2s can be mobilized by helminth or fungal infections to develop into nILC2-like or ILC3-like cells, with ROR*γ*t expression and IL-17 production (Huang et al., 2015; Huang and Paul, 2016; Huang et al., 2018). iILC2s are indeed transient ILC progenitors with multipotency, acting in corresponding immune responses. In addition, Cai et al. reported the ST2^+^ nILC2 population which they defined as ILC2_17_s, were highly pathogenic and critical source of IL-17 in lung inflammation, in response to IL-33 (Cai et al., 2019). The evidence above reveals specific activation factors (IL-25 and IL-33) and function effector (IL-17) of ILC3-like cells.

## ILC3s in Infectious Disease

Here, we mainly discuss how pulmonary ILC3s protect against the challenge of virus, bacteria, parasites, and fungi, as well as the mechanisms underlying the ILC-mediated immunity (**Table 1**).

**Table 1:** ILC3s-mediated effector functions and the activation signals in infection with different lung-invading pathogens.

References	Pathogens		Involved innate immune cell population	Species	Tissue localization	Inducing signals	Effector cytokines
Ivanov et al., 2013	Viruses	H3N2	ILC3s, iNKT cells, *γδ* T cells	Mouse	Lung	Absent	IL-22
Van Maele et al., 2014	Bacteria	*Streptococcus pneumoniae*	ILC3s	Mouse	Lung	IL-23	IL-17A, IL-17F, IL-22
Trevejo-Nunez et al., 2016		*Streptococcus pneumoniae*	ILC3s, *γδ* T cells	Mouse	Lung	IL-23, IL-1β	IL-22
Gray et al., 2017		*Streptococcus pneumoniae*	ILC3s	Mouse	Lung, BALF	Absent	IL-22
				Human	BALF	Absent	IL-22
Oherle et al., 2020		*Streptococcus pneumoniae*	ILC3s	Mouse	Lung, bone marrow, small intestine	Absent	IL-22
				Human	BALF	Absent	IL-22
Xiong et al., 2016		*Klebsiella pneumoniae*	ILC3s	Mouse	Lung	TNF	IL-17A
Bayes et al., 2016a		*Pseudomonas aeruginosa*	ILC3s, *γδ* T cells, B1 cells	Mouse	Lung, mediastinal lymph node	Absent	IL-17A
Muir et al., 2016		*Pseudomonas aeruginosa*	ILC3s, *αβ* T cells	Mouse	Lung	IL-23, IL-2	IL-17A
Ardain et al., 2019		*Mycobacterium tuberculosis*	ILC3s	Mouse	Lung	IL-23	IL-22, IL-17
				Human	Lung, blood	IL-23	IL-22, IL-17
Tripathi et al., 2019		*Mycobacterium tuberculosis*	ILC3s	Mouse	Lung	Absent	IL-22
Mear et al., 2014	Fungi	*Candida albicans*	LTi cells (CD25^+^ ILCs), NK cells, NKT cells	Mouse	Lung	Absent	IL-22, IL-17
Reeder et al., 2018		*Aspergillus fumigatus*	ILC3s, iNKT cells, *γδ* T cells	Mouse	Lung	Absent	IL-22
Huang et al., 2015	Helminths	*Nippostrongylus brasiliensis*	iILC2s	Mouse	Lung	IL-25	IL-17
Huang et al., 2018		*Nippostrongylus brasiliensis*	iILC2s	Mouse	Lung, liver, intestine, spleen, mesenteric lymph nodes	IL-25	IL-17A

NK, natural killer (cells); iNKT, invariant natural killer T (cells); iILC2s, inflammatory ILC2s; BALF, bronchoalveolar lavage fluid.

### Viruses

An increase in IL-17A and IL-17F were detected as early as 2 days post-infection in lung tissue of mice challenged with influenza A/PR/8/34 (H1N1), resulting in excessive neutrophil recruitment and lung injury (Crowe et al., 2009). IL-22 also has crucial functions in the regulation of immunity and tissue repair at barrier surfaces during influenza infection (Sonnenberg et al., 2011). In the early phase of influenza A virus (IAV) infection, ROR*γ*t-expressing innate immune cells such as ILCs, *αβ* and *γδ* T cells, can enhance expression of IL-22 transcripts (Ivanov et al., 2013). IL-22-deficient mice have exacerbated lung injuries, weakened lung function, and decreased airway epithelial integrity during sublethal IAV infection (Ivanov et al., 2013; Pociask et al., 2013). Both IL-17 and IL-22 are involved in the early immune response to pulmonary influenza infection, suggesting a potential role for ILC3s, especially prior to the onset of adaptive immune response. Effects of anti-IL-17 treatment has been proved in ameliorating acute IAV-caused lung injury (Li et al., 2012), whereas anti-IL-22 neutralization had no impact on the respiratory tissue remodeling, lung function, weight loss, and mortality, though the virus titers were reduced (Guo and Topham, 2010; Monticelli et al., 2011). These results negate the hypothesis that IL-22 is critical for influenza virus controlling, and additional studies are necessary to investigate its role.

### Respiratory Bacterial Infections

In murine with various bacterial infections, different cytokines play a dominant role. Early production of IL-17 and IL-22 is a critical factor in neutrophil recruitment as well as in host defense against pathogens *via* the effect on the induction of antimicrobial peptide expression (Ye et al., 2001; Liang et al., 2006; Aujla et al., 2008; Bayes et al., 2016a; Trevejo-Nunez et al., 2016; Valeri and Raffatellu, 2016). Flagellin, a bacterial protein, can enhance their production *via* DC activation in the spleen and mucosa in mice *via* stimulating Toll-like receptor 5 (TLR5) (Van Maele et al., 2010; Kinnebrew et al., 2012). Gray et al. and Van Maele et al. have demonstrated respectively intestinal DCs (Gray et al., 2017) and bone marrow-derived DCs (Van Maele et al., 2014) could drive ILC3s to the lungs. Disruption of postnatal commensal colonization or selective depletion of DCs interrupted lung IL-22^+^ ILC3 migration and rendered the newborn mice more susceptible to *S. pneumoniae* infection, which was reversed by transfer of commensal bacteria after birth (Gray et al., 2017). These findings supported research development of immune-based therapies against respiratory infection targeting TLR5-DCs-ILC3s-IL-22. The effect of flagellin intervention was confirmed showing protection against lethal *Streptococcus pneumoniae* (*S. pneumoniae*) lung infection by promoting IL-22 production (Van Maele et al., 2014). Trevejo-Nunez et al. found that IL-22 systematically administered to *S. pneumoniae*-infected wild-type mice decreased bacterial load in the lung and liver 24 h post-infection by potentiating Complement component 3 (C3) opsonization on bacterial surfaces through the increase of hepatic C3 expression (Trevejo-Nunez et al., 2016). This is the first study confirming IL-22 induction in the lung after a pneumococcal lung infection and exploring its connection with liver. The authors suggested that IL-22:Fc, a recombinant fusion protein containing human IL-22 and immunoglobulin G2 (IgG2)-Fc might be a novel adjunct therapy for severe pneumococcal infection. The relevant clinical trial results are worth anticipating.

The IL-17 family cytokines are found to be important in mediating a neutrophil response and inducing a variety of innate antimicrobial peptides (Iwakura et al., 2008). Several studies have reported ILC3s as the major IL-17A producers in various pulmonary bacterial infections (Bayes et al., 2016a; Muir et al., 2016; Xiong et al., 2016). In an experimental model of acute respiratory distress syndrome (ARDS) induced by *Pseudomonas aeruginosa* (*P. aeruginosa*) infection or aerosolized lipopolysaccharide (LPS), pulmonary ILC3s play a crucial role in recruiting neutrophils to the lung, associated with myeloperoxidase (MPO), IL-6 and the IL-17-dependent neutrophil chemoattractant MIP-2 (Muir et al., 2016). Xiong et al. have demonstrated inflammatory monocytes (IMs) promoted lung ILC3 recruitment and IL-17A production in *Klebsiella pneumoniae* (*K. pneumoniae*)-infected mice (Xiong et al., 2016). Depletion of ILCs from Rag2^−/−^ mice resulted in reduced bacterial clearance, and anti-IL-17A treatment or infection of IL-17A^−/−^ mice led to higher bacterial burden and mortality (Xiong et al., 2016). Bayes et al. confirmed IL-17 cytokine signaling in mouse survival and prevention of chronic infection in mice inoculated with two different strains of *P. aeruginosa* (Bayes et al., 2016a); contrarily, they also drew a conclusion that IL-22 was dispensable in protection against *P. aeruginosa* (Bayes et al., 2016b). The above findings mainly stressed IL-17A production of ILC3s instead of IL-22, in host defense against these infections, suggesting possible immunological differences between various pathogens.

Tuberculosis (TB) is one of the leading causes of death by an infectious disease worldwide. While various immune cells including CD4^+^ T cells, alveolar macrophages (AMs), and epithelial cells are thought to be the main players in immune responses to Mtb, the involvement of ILCs remains unknown. Recently, Ardain et al. first revealed the early protective effect of ILC3s on the immune response to Mtb infection (Ardain et al., 2019). They demonstrated that ILC3s accumulated rapidly within Mtb-infected murine lungs and coincided with the recruitment of AMs, promoting Mtb control (Ardain et al., 2019). In another study, IL-22 is mainly produced by two subsets of ILCs, lymphoid tissue inducer (LTi) cells (50%) and NCR^+^ ILC3s (18%), and is significantly reduced in Mtb-infected mice with type 2 diabetes mellitus (T2DM) than in Mtb-infected mice without T2DM (Tripathi et al., 2019). IL-22 is protective against this hypervirulent strain Mtb HN878 in mice (Treerat et al., 2017) and reduces the severity of lung inflammation and neutrophil-mediated lung epithelial cell injury by inducing the expression of anti-apoptotic proteins Bcl2 and Mcl1 and enhancing the production of antimicrobial peptides (Tripathi et al., 2019). The authors confirmed the roles of IL-22-producing ILC3s by recombinant IL-22 treatment and adoptive transfer of ILC3s, which prolonged the survival of T2DM mice infected with Mtb. This study brings great clinical significance in patients with T2DM, since individuals are susceptible to various infections, and Mtb is one of the most common pathogens. Further understanding of potential mechanisms of the ILC3s-IL-22 pathway in human studies may help to treat T2DM patients with Mtb infection.

Most of the research studies focus on murine infections, whereas human studies are relatively rare. ILC3s are critical for lung defense against bacterial pneumonia in the neonatal period, the leading cause of death in newborn infants (Gray et al., 2017). The numbers of lung IL-22^+^ ILC3s were significantly decreased in the BALF of human newborns exposed to prolonged duration of antibiotics (ABX), suggesting new therapeutic agent development to mitigate the risk associated with early life ABX exposure in children. Signals that guide lung ILC3 development in newborns remain unclear until Oherle et al. showed that insulin-like growth factor 1 (IGF1) produced by alveolar fibroblasts instructed expansion and maturation of pulmonary ILC precursors, linking postnatal lung growth with development of pulmonary ILC3s (Oherle et al., 2020). This study laid a foundation for further exploration of lung ILC3s in human pulmonary host defense and mucosal immunity, especially in newborns and children. Moreover, Ardain et al. recently showed that C-X-C motif chemokine receptor 5 (CXCR5) on circulating ILC3s was upregulated in patients with Mtb, accompanied by increased levels of its ligand C-X-C motif chemokine ligand 13 (CXCL13) in the plasma (Ardain et al., 2019). The expansion of ILC3s and IL-17 and IL-22 production are essential factors of lung CXCL13, early innate immunity, alveolar macrophage accumulation, and protective lymphoid follicle-containing granulomas formation (Okamoto Yoshida et al., 2010; Ardain et al., 2019). The authors confirmed the protective roles of ILC3s, consistent with their murine models.

### Respiratory Fungal Infections

*Candida albicans* (*C. albicans*) and *Aspergillus fumigatus* (*A. fumigatus*) are representatives of the most common fungal infections. IL-17-mediated effector response is a crucial factor in host defense against mucosal fungal infection and pathogen clearance (Gladiator et al., 2013; Sparber and LeibundGut-Landmann, 2015). In mice upon oropharyngeal candidiasis infection, IL-17A and IL-17F were produced promptly, mainly by ILCs in the oral mucosa (Gladiator et al., 2013). Depletion of ILCs by antibodies in Rag1-deficient mice or ROR*γ*t deletion resulted in a complete failure to infection control (Gladiator et al., 2013). In another study, the role of IL-22-producing ILCs was stressed. *C. albicans* airway exposure led to the recruitment and activation of NK cells, ILCs, macrophages, and DCs, of which ILCs were the only cellular source of IL-22 (Mear et al., 2014). IL-22 primes a protective neutrophil-independent response by increasing expression of antimicrobial peptides *β*-defensin-2, -3 and -4 with a synergistic effect on cathelicidin (LL-37) (Mear et al., 2014). However, it is a pity that the whole population of ILCs is marked in flow cytometry in these two studies instead of an exact definition of ILC3s, although ILC3s are considered to be the only ILC subset with a capability of IL-17 production. As we know, specific deletion of ILC3s could not be achieved by using anti-CD90.2 antibodies. In the first study, RORc^−/−^ mice are not only deficient in ILC3s but also CD4^+^ Th17 cells and CD8^+^ cytotoxic T (Tc) cells, so it’s hard to distinguish the effects of these groups from the models.

Humans with suppressed immune systems or genetic immunodeficiencies are constantly exposed to the opportunistic infection with mold *A. fumigatus*. In murine models, Dectin-1-deficient mice have multiple defects in host defense against acute exposure to *A. fumigatus*, which are associated with a near total loss of lung IL-22 production (Werner et al., 2009; Gessner et al., 2012; Lilly et al., 2012). Reeder et al. identified innate immune cells invariant natural killer T (iNKT) cells, *γδ* T cells, and ILC3s as major sources of IL-22 post-exposure with acute *A. fumigatus* infection (Reeder et al., 2018). Since the development and maintenance of these cells usually require common *γ*-chain cytokines, the authors explored the roles of IL-7, IL-21, and IL-15 in IL-22 induction (Reeder et al., 2018). They found IL-21 was responsible for maintaining ILC3s and fungal clearance; however, the absence of IL-7, IL-21, or IL-15R signaling had no impact on neutrophil recruitment (Reeder et al., 2018). Although inconsistent results were observed, these results provided new insights into how the IL-22 response in the lung is shaped during fungal exposure.

There is a lack of research on lung ILC3s and fungal infections in human. The study of patients with various primary autosomal immunodeficiencies has suggested that IL-17A, IL-17F, as well as the frequently associated IL-22, are major cytokines for mucocutaneous immunity to *C. albicans* (Cypowyj et al., 2012). Research studies linking the cytokine-producing ILC3s to patients with fungal infections and the potential pathogenesis are warranted.

### Helminthic Lung Infections

Helminth larvae migrate to the lung to molt, inducing hypersensitive reaction and resulting in hemorrhage and acute lung injury. Studies have demonstrated that upon the rodent hookworm *Nippostrongylus brasiliensis* (*N. brasiliensis*), type 2 responses were driven by ILC2-derived cytokines IL-4, IL-5, IL-9, and IL-13 in an IL-33-dependent manner (Bando et al., 2013; Hung et al., 2013; Turner et al., 2013). Huang et al. found that inflammatory ILC2s (iILC2s) promoted anti-helminth defense and tissue repair after *N. brasiliensis*-induced lung inflammation (Huang et al., 2015; Huang et al., 2018), as discussed above. In helminthic and fungal immunity, iILC2s were transient progenitors of ILCs mobilized by infection that develop into nILC2s-like cells or ILC3-like cells, producing IL-17 instead of IL-13 (Huang et al., 2015). They also found that iILC2s arose from resting ILC2s residing in the intestinal lamina propria, which migrated to the lung through sphingosine 1-phosphate (S1P)-mediated chemotaxis (Huang et al., 2018). Type 2 immune responses are commonly considered to dominate the immune microenvironment of parasitic infection (Hotez et al., 2008; Hung et al., 2013). This ILC2 expansion and migration to effector sites indicate that ILCs provide both local and distant tissue protection during infection. Explicating ILC plasticity may help us explore the mechanism of innate immune response, as well as develop or improve options for novel therapies.

## ILC3s in Inflammatory Airway Diseases

Most studies have focused on ILC2s, emphasizing their importance in limitation to immunological stimuli exposure, maintenance of the epithelial barrier, and involvement in allergic inflammatory diseases. In recent years, ILC3s have also been found to play a key role in the orchestration of airway inflammatory diseases (**Table 2**).

**Table 2:** ILC3s-mediated effector functions and the activation signals in airway inflammation.

References	Diseases		Involved innate immune cell population	Species	Tissue localization	Inducing signals	Effector cytokines
Kim et al., 2014	Asthma	Obesity-related asthma	ILC3s	Mouse	Lung, white adipose tissue	IL-1β	IL-17A
				Human	BALF	IL-1β (IL-2, IL-7)	IL-17
Everaere et al., 2016		Obesity-related asthma	ILC3s	Mouse	Lung, blood, adipose tissue	IL-1β	IL-17
Hekking et al., 2018		Adult-onset severe asthma	ILC3s	Human	Lung, sputum, nasal brushings, endobronchial brushings	Absent	Absent
Komlosi et al., 2018		Allergic asthma	ILC3s	Human	Blood, tonsil	IL-23	IL-22
Cai et al., 2019		Papain-induced asthma	ILC3-like ILC2_17_s	Mouse	Lung, intestine, bone marrow	IL-33	IL-17
Warren et al., 2019		ODE-OVA-induced asthma	ILC3s, NK cells	Mouse	Lung, BALF	Absent	Absent
Kim et al., 2019		Non-eosinophilic asthma	ILC3s	Mouse	Lung	IL-2, IL-12, IL-23	IL-17A
				Human	Sputum	Absent	Absent
Lee et al., 2020		Non-allergic eosinophilic asthma	ILC3s	Mouse	Lung	IL-23	IL-17A
Michaudel et al., 2020		Ozone-induced asthma	ILC3s, NK cells, *αβ* T cells, *γδ* T cells	Mouse	Lung	Absent	IL-22, IL-17A
De Grove et al., 2016	COPD	–	ILC3s	Human	Lung	Absent	IL-22, IL-17A, GM-CSF
Yanagisawa et al., 2017		–	ILC3s, *γδ* T cells	Mouse	Lung	IL-1β	IL-17A
Shikhagaie et al., 2017		–	ILC3s	Human	Lung, blood, skin, gut, mesenteric lymph node, spleen, post-natal thymus	IL-2, IL-1β, IL-23	IL-22
Suzuki et al., 2017		–	LTi cells, ILC1s, NK cells	Human	Lung	Absent	Absent
Starkey et al., 2019		–	ILC3s, NKT cells, *γδ* T cells	Human	Lung	Absent	IL-22
		–		Mouse	Lung	Absent	IL-22
Blomme et al., 2018		–	ILC3s	Mouse	Lung, BALF	Absent	IL-17

NK, natural killer (cells); OVA, ovalbumin; ODE, organic dust extract; BALF, bronchoalveolar lavage fluid; COPD, chronic obstructive pulmonary disease; GM-CSF, granulocyte-macrophage colony-stimulating factor.

### Asthma

Asthma is a highly heterogeneous chronic airway disease subdivided into several subsets. Type 2 (T2)-high (or Th2) asthma and T2-low (or non-Th2) asthma are considered to be two major subtypes (Huang et al., 2019; Kuruvilla et al., 2019). Non-Th2 asthma includes neutrophilic asthma, paucigranulocytic asthma, and obesity-related asthma (Huang et al., 2019). There are a variety of experimental asthma models in airway inflammation, with distinct underlying pathogenesis.

In 2011, Taube et al. found that the major sources of IL-22 production were ILCs rather than Th cells, in an allergic asthma model induced by ovalbumin (OVA) (Taube et al., 2011). IL-22 has a prominent protective impact on airway inflammation and tissue damage upon airway challenge, and mice treated with IL-22 before antigen challenge display significant amelioration of airway constriction and inflammation by reducing expression of CCL17 (TARC) and IL-13 (Taube et al., 2011). Similarly, it is a pity that ILC3s are not well-defined in this study, but the authors put forward a novel therapeutic approach with rIL-22 for patients with allergic asthma.

In the lungs, the role of inflammation in obesity has been controversial, due to limited reports of correlation between airway eosinophils and body mass index (BMI), until the neutrophilic phenotype in obese asthmatics was reported (Scott et al., 2011; Telenga et al., 2012). Kim et al. demonstrated the role of CCR6^+^ IL-17A-producing ILC3s as well as NLRP3-IL-1β signaling in lung inflammation for the first time, through a mouse model with an HFD-induced asthma (Kim et al., 2014). Although previous studies in allergic asthma have conflicting results (Kool et al., 2011; Marichal et al., 2011; Allen et al., 2012), the pathological role of NLRP3 in airway neutrophilic inflammation is supported in a toluene diisocyanate (TDI)-induced asthma model recently (Chen et al., 2019). In-house dust mite (HDM)-challenged mice, total cytokine-expressing ILC2s and ILC3s in the lung were increased in the obese group compared to the lean group, resulting in exacerbation of AAI (Everaere et al., 2016). There are differences between these two models; for example, the former is obesity-induced nonallergic asthma while the latter is HDM-induced allergic asthma with obesity. Collectively, obesity-associated asthma is facilitated by ILC3s-mediated inflammation.

More recently, research studies have become more and more popular focusing on ILC3s and asthma. Lee et al. reported a murine model of non-allergic eosinophilic asthma (NAEA) developed by administration of recombinant IL-23 (rIL-23) plus a low dose non-specific airway irritant (polyI:C or DEPs) without allergen (Lee et al., 2020). Administration of the agents induced the release of innate cytokines from the airway epithelium, including IL-33, thymic stromal lymphopoietin (TSLP), and IL-1β, leading to ILC2s and ILC3s activation in the lung (Lee et al., 2020). One limitation to note is that the effects of ILC2s and ILC3s can’t be distinguished in this model since it is not easy to develop mice with specific deletion of ILC2s. In OVA-organic dust extract (ODE)-treated mice, modeling an occupational exposure on asthmatics, NKp46^+^ ILC3s were increased in the BALF as compared to mice treated with ODE or OVA alone (Warren et al., 2019). ILC3s were not recruited upon ODE exposure alone, suggesting essential involvement of mixed or “two-hit” signals to engage ILC3s. CCL2 (monocyte chemotactic protein), CCL3 (macrophage inflammatory protein-1*α*), and CXCL1/CXCL2 (neutrophil chemoattractants) were elevated in response to ODE exposure (Warren et al., 2019). In chronic ozone exposure-induced airway inflammation and hyperreactivity, recruitment of IL-22-producing cells including ILC3s, NK cells, and T cells was modulated by AhR, resulting in the inflammation control (Michaudel et al., 2020). As far as we know, IL-22 usually plays an anti-inflammatory and protective role pathogen challenge, as mentioned above. Whether IL-22 has a similar or contrary effect on infectious and non-infectious inflammation is needed for further research.

Similar to the findings in helminthic lung infections, ST2^+^ nILC2s (called ILC2_17_s) are defined in the lung inflammation induced by papain or IL-33, which possesses some characteristics of ILC3s, for example, AhR expression and IL-17 production (Cai et al., 2019). IL-33 worked in synergy with leukotrienes and promoted IL-17 production in ILC2_17_s, signaling through nuclear factor of activated T-cell (NFAT) activation or the hematopoietic compartment of MyD88 (Cai et al., 2019). However, different from the previous studies, the data for the first time demonstrated the pathogenic role of lung-resident nILC2s in lung inflammation, instead of iILC2s at other sites elicited by helminth infection or IL-25 treatment (Huang et al., 2015; Huang and Paul, 2016; Huang et al., 2018). It might be interesting and prospective to explore the relationship and signal pathway between these two subsets.

Experimental murine models can hardly exactly model asthma in patients, might leading to different pathogenesis. For example, OVA, widely used to elicit allergic asthma, is not a natural allergen. Thus, more human research studies are warranted to verify the results of murine models. Kim et al. found IL-17^+^ ILC3s were also elevated in the BALF of patients with asthma compared to healthy controls (Kim et al., 2014). However, as the number of BALF ILC3s varied considerably in the 10 patients examined (Kim et al., 2014), clinical studies with a larger number of patients are required. Recently, using transcriptomic profiles, Hekking et al. have identified highly enriched ILC3 gene signatures and the inflammatory pathways involving eosinophils and mast cells in the airway secretions of patients with adult-onset severe asthma (Hekking et al., 2018). This is the first study to reveal a correlation between ILC3s and adult-onset (severe) asthma and indicates a particular involvement of mast cells; however, there is no correction for cell counts (*e.g.*, eosinophils) in the analysis, which may result in underdetection of actual pathways. Patients with eosinophilic asthma and non-eosinophilic asthma have different profiles of ILC-macrophage interactions; in non-eosinophilic asthma, ILC1s and ILC3s promote M1 macrophage polarization by secreting IFN-*γ* or IL-17A (Kim et al., 2019). All these findings support the roles of ILC3s in human asthma; however, few molecular links have been discussed in these studies. Interestingly, Komlosi et al. reported that ILC3s were relatively reduced in tonsil tissue of patients with allergic disease as well as in peripheral blood of allergic asthmatic patients compared with that of healthy controls (Komlosi et al., 2018). ILC3s help B-cell proliferation and differentiation of functional itBreg cells with IL-10 secretion and PD-L1 expression, in a CD40 ligand (CD40L)- and B cell-activating factor receptor (BAFF)-dependent manner (Komlosi et al., 2018). They speculated the reduced ILC3 levels could contribute to the maintenance of insufficient Breg cell-mediated immune tolerance in patients with allergic asthma.

### COPD

Chronic obstructive pulmonary disease (COPD) is characterized by airflow limitation, lung destruction, and increased neutrophilic infiltration associated with Th17-related cytokine expression (Di Stefano et al., 2009). It is commonly triggered by noxious stimuli such as cigarette smoke (CS). In a murine model of COPD, all ILC subsets including T-bet^+^ ILC1s, ST2^+^ ILC2s, and ROR*γ*t^+^ ILC3s were increased markedly in the smoking group associated with the level of IFN-*γ* or IL-17, indicating the prominent role of ILC1s and ILC3s (Blomme et al., 2018). ILC3s are one of the important sources of IL-17A (Yanagisawa et al., 2017) and IL-22 (Starkey et al., 2019) in murine COPD. Neutralization of IL-17A/IL-17RA axis protects against airway inflammation and fibrosis, although the total number of ILC3s is not significantly different between CS+polyinosinic:polycytidylic acid (PIC)-exposed and room air-exposed mice (Yanagisawa et al., 2017). Surprisingly, IL-22-deficient mice with CS exposure had improved lung function and reduced pulmonary neutrophils, suggesting an impact of IL-22 on impairing lung function and promoting inflammation (Starkey et al., 2019). However, treatment targeting IL-22 signaling on COPD should be carefully assessed, which possibly increases the risk of exacerbations due to its central role in pathogen clearance.

Although direct evidence in mechanisms linking ILC3s to COPD is limited, the cell populations have been identified in lung tissues of COPD patients (Di Stefano et al., 2009; De Grove et al., 2016; Suzuki et al., 2017). IL-17A^+^, IL-22^+^, and IL-23^+^ immunoreactive cells are increased in bronchial biopsies of patients with stable COPD compared to control subjects (Di Stefano et al., 2009). A tendency to a higher frequency of the subset NCR^−^ ILC3s was observed in the lungs of COPD patients (De Grove et al., 2016). Suzuki et al. first showed enriched expression profiles of LTi cells and NK cells, and ILC1s, associated with T cell and B cell infiltration in lung tissues of patients with severe COPD, using gene set enrichment analysis (Suzuki et al., 2017). However, these results might be considered preliminary due to its coming from only four lungs of severe COPD (GOLD 4) patients, and their findings would be more robust and representative if the number of cases was greater and patient population was more extensive. Shikhagaie et al. found that neuropilin-1 (NRP1)-expressing ILC3s (LTi-like cells) were present in human fetal tissues and adult lymphoid tissues, as well as in the lungs of smokers and COPD patients (Shikhagaie et al., 2017). NRP1^+^ ILC3s produce IL-22, and upregulate receptor for vascular cell adhesion molecule (VCAM1) and receptor for intracellular adhesion molecule (ICAM1) on mesenchymal stromal cells (MSCs) (Shikhagaie et al., 2017). Their results implied a role of ILC3s in the initiation of ectopic pulmonary lymphoid aggregates and airway remodeling in COPD.

## Concluding Remarks

While research studies on ILC3s have become a focus in the intestinal immunity (Withers and Hepworth, 2017; Pantazi and Powell, 2019), the biology and function of pulmonary ILC3s in the pathogenesis of respiratory diseases remain elusive. In this review, we have mainly discussed how pulmonary ILC3s act on protection against pathogen challenge and pulmonary inflammation. As is highlighted here, many studies have established the importance of ILC3s in regulating epithelial function, mucus production, and tissue repair *via* the rapid secretion of IL-17 and IL-22. Targeting ILC3s and their underlying signaling pathways may contribute to provide a new field for potential immune-based therapies on respiratory infection and airway inflammation.

However, relatively few research studies focus on how the inducing factors signal on lung ILC3s. The cellular and molecular communication in host–pathogen interactions and the expansion and/or recruitment process of ILC3s, especially in human still remain poorly understood. As for the major cytokines produced by ILC3s, IL-17, and IL-22 are mainly reported as protective effectors in host defense during infections, whereas proinflammatory and pathogenic effects tend to dominate in non-infectious inflammation. Proinflammatory and tissue-protective properties of IL-17A and IL-22 might coexist in the same model, and studies have revealed IL-17A/IL-22 collaboration promoted IL-22 proinflammatory activity (Andoh et al., 2005; Boniface et al., 2005; Sonnenberg et al., 2010b). More studies are needed to compare the dominant roles of ILC3s and latent mechanisms in different lung immunity.

In addition, the definitions and markers of ILC3s are not exactly the same in various studies, which means the researchers are not investigating the specific populations. This is not only for ILC3s, but also for other ILC groups, which might also be one of the reasons for the different effects between studies. Therefore, careful evaluation and comparison are needed before referring to the results for future researchers.

Moreover, there are various discrepancies in the characterization between human and murine (Annunziato et al., 2015; Vivier et al., 2018; Shao et al., 2019; Yudanin et al., 2019), for example, the distinct origin, differentiation, distribution, activation and functions of lung ILC3s, the anatomic and physiological differences in airways, as well as the divergent pathophysiologic changes of diseases. Although many research studies have revealed the role of ILC3s and the efficacy of treatment targeting the related pathway in murine models, there is still a long way to go to verify the results in patients.

## Author Contributions

All authors listed have made a substantial, direct, and intellectual contribution to the work and approved it for publication. DY and XG have contributed equally to this work.

## Funding

This study was supported by the National Natural Science Foundation of China (Grant No. 81770035).

## Conflict of Interest

The authors declare that the research was conducted in the absence of any commercial or financial relationships that could be construed as a potential conflict of interest.

## Abbreviations

Th, T helper cells; IL-17A, interleukin 17A; ILCs, innate lymphoid cells; ILC3s, group 3 innate lymphoid cells; Inkt, invariant natural killer T (cells); NK, natural killer (cells); LTi, lymphoid tissue inducer (cells); RORγt, retinoic acid receptor-related orphan receptor γt; AhR, Aryl hydrocarbon receptor; GM-CSF, granulocyte-macrophage colony-stimulating factor; NCR, natural cytotoxicity receptor; CCR6, C-C motif chemokine receptor 6; IAV, influenza A virus; Mtb, Mycobacterium tuberculosis; DCs, dendritic cells; TLR, Toll-like receptor; ARDS, acute respiratory distress syndrome; LPS, lipopolysaccharide; AMs, alveolar macrophages; CXCR5, C-X-C motif chemokine receptor 5; CXCL13, C-X-C motif chemokine ligand 13; T2DM, type 2 diabetes mellitus; ABX, antibiotics; IGF1, insulin-like growth factor 1; iILC2s, inflammatory ILC2s; nILC2s, natural ILC2s; T2, type 2; HFD, high-fat diet; AHR, airway hyperreactivity; OVA, ovalbumin; NFAT, nuclear factor of activated T-cells; NAEA, nonallergic eosinophilic asthma; ODE, organic dust extract; BALF, bronchoalveolar lavage fluid; COPD, chronic obstructive pulmonary disease; IMs, inflammatory monocytes; MPO, myeloperoxidase; S1P, sphingosine 1-phosphate; AAI, allergic airway inflammation; CS, cigarette smoke; VCAM1, vascular cell adhesion molecule; ICAM1, receptor for intracellular adhesion molecule; MSCs, mesenchymal stromal cells; TNF, tumor necrosis factor; ASMs, airway smooth muscles; M ø, macrophages; BAFF, B cell-activating factor receptor.
