# Supplementary material for: The Role of Group 3 Innate Lymphoid Cells in Lung Infection and Immunity
Source: Front Cell Infect Microbiol. 2021 Feb 25;11:586471. doi: 10.3389/fcimb.2021.586471 (PMC7947361; doi:10.3389/fcimb.2021.586471)
Supplement: Supplementary file 1 [file DataSheet_1.docx]

Supplementary Figure 1 Schematic representation of ILC development.


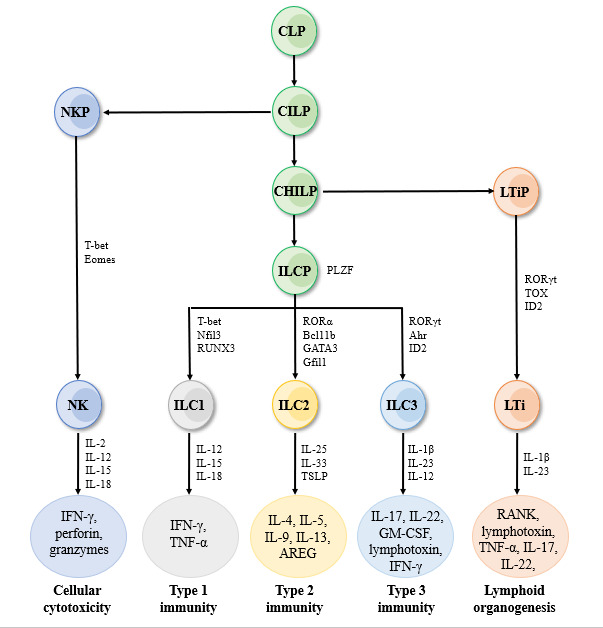


ILC development is shown, mainly based on mouse ILC differentiation paths. ILCs, are classified into five distinct subsets on the basis of the transcription factor involved and unique spectrum of cytokines. Common lymphoid progenitors (CLPs) differentiate into common innate lymphoid progenitors (CILPs), and ILCs develop from CILPs. CILPs give rise to common helper innate lymphoid progenitors (CHILPs) or NK cell precursors (NKPs), in which CHILPs differentiate into lymphoid tissue inducer progenitors (LTiPs) and promyelocytic leukemia zinc finger (PLZF)-expressing innate lymphoid cell precursors (ILCPs). ILCPs differentiate into ILC1s, ILC2s, or ILC3s, LTiPs into LTi cells, and NKPs into NK cells, depending on the expression of the indicated transcription factors involved in their development such as T-box transcription factor (T-bet), Eomesodermin (Eomes), nuclear factor, IL-3 regulated (NFIL3), runt-related transcription factor 3 (RUNX3), retinoic acid receptor-related orphan receptor α (RORα), B cell lymphoma/leukemia 11B (Bcl11b), growth factor independent 1 (Gfi1), GATA binding protein 3 (GATA3), inhibitor of DNA binding 2 (ID2), retinoic acid receptor-related orphan receptor γt (RORγt), Aryl hydrocarbon receptor (AhR), thymocyte selection-associated high mobility group box protein (TOX).
